# Brain-derived Neurotrophic Factor in Megakaryocytes*^♦^

**DOI:** 10.1074/jbc.M116.720029

**Published:** 2016-03-22

**Authors:** Pedro Chacón-Fernández, Katharina Säuberli, Maria Colzani, Thomas Moreau, Cedric Ghevaert, Yves-Alain Barde

**Affiliations:** From the ‡ School of Biosciences, Cardiff University, Cardiff CF10 3AX, Wales and; the § Department of Haematology, University of Cambridge, Cambridge CB2 0PT, United Kingdom

**Keywords:** blood, brain-derived neurotrophic factor (BDNF), neurobiology, neurochemistry, neurotrophin

## Abstract

The biosynthesis of endogenous brain-derived neurotrophic factor (BDNF) has thus far been examined in neurons where it is expressed at very low levels, in an activity-dependent fashion. In humans, BDNF has long been known to accumulate in circulating platelets, at levels far higher than in the brain. During the process of blood coagulation, BDNF is released from platelets, which has led to its extensive use as a readily accessible biomarker, under the assumption that serum levels may somehow reflect brain levels. To identify the cellular origin of BDNF in platelets, we established primary cultures of megakaryocytes, the progenitors of platelets, and we found that human and rat megakaryocytes express the *BDNF* gene. Surprisingly, the pattern of mRNA transcripts is similar to neurons. In the presence of thapsigargin and external calcium, the levels of the mRNA species leading to efficient BDNF translation rapidly increase. Under these conditions, pro-BDNF, the obligatory precursor of biologically active BDNF, becomes readily detectable. Megakaryocytes store BDNF in α-granules, with more than 80% of them also containing platelet factor 4. By contrast, BDNF is undetectable in mouse megakaryocytes, in line with the absence of BDNF in mouse serum. These findings suggest that alterations of BDNF levels in human serum as reported in studies dealing with depression or physical exercise may primarily reflect changes occurring in megakaryocytes and platelets, including the ability of the latter to retain and release BDNF.

## Introduction

BDNF^2^ is a secretory protein regulating the development and function of neural circuits (1, 2). The functional relevance of BDNF in humans is firmly established following the discovery of polymorphisms and loss-of-allele mutations associated with deficits ranging from subtle memory alterations (3) to severe symptoms early in life (4). The cDNA sequence of *BDNF* predicts that like other cystine-knot proteins, BDNF is first synthesized as a precursor protein, referred to as pro-BDNF ensuring the proper formation of disulfide bridges and of a biologically active, mature neurotrophin (5, 6). Numerous experiments with various artificial expression systems have confirmed this view, in line with the results of early experiments with nerve growth factor (7). So far, only a very small number of studies have been performed addressing the question of the biosynthesis, storage, and secretion of endogenous BDNF (8, 9). As a result of the scarcity of the protein in neurons, most studies used instead overexpression paradigms, leading to uncertainties as to whether the processing of pro-BDNF takes place within neurons or also in the extracellular space following the secretion of pro-BDNF. Human platelets contain between 100–1,000-fold more BDNF than brain tissue when brain and platelets are compared on a protein basis (10–12). As it appears unlikely that the biosynthesis of BDNF takes place in platelets, we established primary cultures of megakaryocytes (Mks), the progenitors of platelets. Beyond questions related to the biosynthesis of endogenous BDNF and to the productive expression of its gene in non-neuronal cells, the question of the origin of BDNF in human blood and serum is of wider interest. Indeed, BDNF levels in human serum are widely used as a biomarker speculated to somehow reflect brain levels. Thus, countless studies have reported decreased BDNF levels in serum in mood disorders, including depression (13), although by contrast physical exercise has been found to increase them (14).

## Experimental Procedures

### 

#### 

##### Reagents

For recombinant proteins, BDNF produced in *Escherichia coli* was from Amgen/Regeneron partners (Tarrytown, NY). Cleavage-resistant mouse pro-BDNF and the BDNF pro-peptide were produced in COS-7 cells transfected with the corresponding cDNA (15). Stem cell factor and thrombopoietin (TPO) were from R&D Systems (Abingdon, UK). Interleukin-1β (IL1-β) was from Miltenyi Biotec (Bisley, Surrey, UK). Human recombinant fibrinogen was from EMD-Millipore (Calbiochem; Darmstadt, Germany). The mouse monoclonal anti-BDNF 3C11 was from Icosagen (Tartu, Estonia). BDNF antibodies directed against mature BDNF recognize the mouse, rat, and human protein with equal affinity as the corresponding amino acid sequence is identical. The mouse monoclonal anti-pro-BDNF H100G was from GeneCopoeia Inc. (Rockville, MD). The monoclonal anti-BDNF#9 was used as described (15). The chicken anti-β-actin Ab13822, the rabbit anti-platelet factor (PF4) Ab129183, and the goat anti-chicken HRP-conjugated secondary antibody Ab97135 were from Abcam (Cambridge, UK). The donkey anti-mouse HRP-conjugated secondary antibody was from Promega (Madison, WI). Alexa Fluor-488 donkey anti-mouse IgG secondary antibody, Alexa Fluor-594 donkey anti-rabbit IgG secondary antibody, and phalloidin-TRITC were purchased from Invitrogen. Hanks' balanced salt solution, Iscove's modified Dulbecco's medium, HEPES, fetal bovine serum (FBS), l-glutamine, and penicillin/streptomycin were from Invitrogen. Cellgro SCGM medium was from Cellgenic (Freiburg, Germany). All other reagents, including thapsigargin and ionomycin, were of analytical grade and were purchased from Sigma (Dorset, UK).

##### Cell Culture and Isolation

Approximately 10-week-old CD1 or C57BL/6 mice and Wistar rats were sacrificed by rising CO_2_ inhalation and blood subsequently drawn by cardiac puncture using acid citric dextrose-B tubes (BD Vacutainer, BD; Plymouth, UK). Femurs and tibias of CD1 or C57BL/6 mice and Wistar rats were removed, and bone marrow was extracted by flushing the bones with Hanks' balanced salt solution containing 0.38% sodium citrate, 1 mm adenosine, 2 mm theophylline, and 5% heat-inactivated FBS. After lysis of the red cells, the suspension was passed through a 40-μm strainer, and the pellet was resuspended in culture medium (Iscove's modified Dulbecco's medium with l-glutamine, 25 mm HEPES, 5% heat-inactivated FBS, 100 units/ml penicillin, and 100 μg/ml streptomycin) containing 25 ng/ml stem cell factor and TPO. After 6–7 days of culture *in vitro*, mature Mks were purified on a 1.5–3% bovine serum albumin gradient and cultured in Cellgro SCGM medium for up to 16 h. Hippocampi and lungs were dissected and kept frozen at −80 °C until use. Human samples, both neonatal cord blood and adult peripheral blood, were obtained after securing informed consent following a protocol approved by the National Research Ethics Service. CD34-positive cells (≥98%) were isolated by magnetic cell sorting, cultured for 10 days in Cellgro SCGM medium containing TPO and IL1-β, and analyzed by flow cytometry with 70–90% CD41a^+^ and 20–60% CD42a^+^ cells representing committed progenitors and mature Mks, respectively. Platelet-rich plasma was obtained from either animal or human samples by centrifugation of acid citric dextrose-B tubes at 200 × *g* for 20 min in the presence of prostaglandin-E1 (1 μm) and apyrase (1 unit/ml) to prevent cellular activation. Platelets were pelleted from platelet-rich plasma by centrifugation at 1,100 × *g* for 10 min and lysed immediately.

##### Western Blot and Densitometric Analyses

Platelets and Mks were lysed for 30 min on ice in a buffer containing 50 mm Tris-HCl, pH 7.4, 150 mm NaCl, 1 mm EDTA, 1% Triton X-100, and 0.2% sodium deoxycholate, supplemented with protease and phosphatase inhibitor mixture mix, 10 μm 1,10-phenanthroline monohydrate, 10 mm 6-aminohexanoic acid and 10 μg/ml aprotinin. After sonication (1 pulse, 50% amplitude), insoluble debris was removed by centrifugation. Proteins were separated on 4–12% NuPAGE gradient gels containing SDS (Invitrogen) and transferred to nitrocellulose membranes using the semi-dry Trans-Blot unit (Bio-Rad, Hertfordshire, UK). To allow the detection of the BDNF pro-peptide (15), the membranes were fixed after transfer with 2.5% glutaraldehyde for 30 min at room temperature and subsequently incubated for 2 h with blocking solution (3% blocking reagent (Invitrogen) and 3% BSA in TBS-T) and then probed overnight at 4 °C with anti-BDNF 3C11 (1:2,000), anti-pro-BDNF H1001G (1:1,000), or chicken anti-β-actin (1:2,000). Binding of primary antibodies was visualized with donkey anti-mouse HRP-conjugated secondary antibodies (1:10,000) or goat anti-chicken HRP-conjugated secondary antibody (1:5,000). Chemiluminescence was developed using the Lumi Glo Reverse Western blotting substrate (Cell Signaling Technology, Danvers, MA). Care was taken in all experiments to ensure that the signal was well within the linear range, and densitometry was carried out using ImageJ software.

##### BDNF ELISA Determinations

BDNF sandwich ELISA was performed using a combination of monoclonal antibodies as described previously (16), with the following modifications. Streptavidin high binding capacity coated white plates (Pierce) were incubated overnight at room temperature with 10 μg/ml mouse anti-BDNF antibody 1 conjugated with biotin (Sulfo-NHS-LC-biotinylation kit EZ-Link, Pierce) in coating buffer (25 mm Tris, 150 mm NaCl, 0.1% BSA, 0.05% Tween 20, pH 7.2). Following an overnight incubation, the plates were washed with 0.1% Tween 20 in PBS (washing buffer) and then blocked for 2 h with 4% BSA in PBS. To each well 150 μl of incubation buffer (0.1 m KH_2_PO_4_, 0.1 m Na_2_HPO4, pH 7.6) were added, followed by 50 μl of sample or BDNF standard. Mk lysates or recombinant BDNF were incubated overnight at 4 °C with 10 μg/ml mouse anti-BDNF antibody 9 conjugated with horseradish peroxidase (activated peroxidase kit, EZ-Link Plus, Pierce). Mk lysates were used at 1:3 in 0.1% Triton X-100 PBS (PBT). To test for a potential release of BDNF from Mks into their incubation medium, purified Mks were cultured on a 24-well plate and incubated for 2 days at 37 °C with 5% CO_2_, in CellGro SCGM medium containing 10 μg/ml horseradish peroxidase-conjugated anti-BDNF antibody 9. The standard curve was established in a parallel plate using various concentrations of recombinant BDNF incubated for 2 days in the same culture medium without cells. The limit of detection is 1.25 pg of BDNF per well. After 2 days, the corresponding media (Mks and standard curve) were collected and incubated for 3 h on a rotating platform followed by a rinse with washing buffer. BM chemiluminescence ELISA substrate POD (Roche Applied Science, Mannheim, Germany) was added, and luminescence was measured with a microplate reader (FLUOstar Omega, BMG labtech). Both standards and samples were determined in triplicate.

##### Immunocytochemistry and Confocal Analysis

Mks were cultured on 200 μg/ml human fibrinogen-coated coverslips for 6 h (mouse and rat Mks) or 36 h (human MKs), fixed for 30 min in 4% paraformaldehyde in PBS, and permeabilized for 15 min at room temperature with 0.5% Triton X-100 in PBS. Mks were then blocked with blocking solution (10% donkey serum in PBT) for 1 h. Primary antibodies were diluted in blocking solution at the following final concentrations/dilutions: 1:500 rabbit anti-PF4 and 10 μg/ml mouse anti-BDNF antibody 9. After 2 h of incubation at room temperature, coverslips were washed three times with PBT and incubated for 1 h with the secondary antibodies used at a 1:500 dilution in blocking solution or phalloidin/TRITC (at 1:50 dilution in blocking solution). After further washing with PBT, labeled coverslips were mounted onto glass slides with DAPI-containing mounting medium. Images were acquired at ×63 magnification using a confocal microscope (LSM 780; Carl Zeiss). Co-localization coefficient between BDNF and PF4 was determined using the Zeiss co-localization coefficient software (ZEN black 2011), which utilizes the Manders overlap coefficient equation to quantify overlapping pixels. At least 15 cells per sample were analyzed.

##### RNA Extraction, Retrotranscription, and Conventional and Real Time Quantitative PCRs

Total RNA was extracted from animal tissue (dissected hippocampi or lungs) using TRIzol reagent (Invitrogen) and cells (mouse, rat, and human MKs) using RNeasy kit (Qiagen, Valencia, CA) according to the manufacturer's instructions, including a DNase treatment. Human RNA samples from hippocampus or lung were obtained from Clontech. cDNA was prepared from 1,250 ng of total RNA using random hexamers (Promega) and SuperScript III first-strand synthesis system (Invitrogen). To analyze expression of mouse, rat, and human *BDNF* exon-specific transcripts, cDNA was amplified with 35–40 cycles of PCR using an annealing temperature of 57–60 °C for all primer combinations (primers listed in Table 1). Real time quantitative PCR was performed on the StepOne system (Applied Biosystems, Invitrogen) using TaqMan probes and primers for mouse, rat, and human *BDNF* exon-specific transcripts or BDNF coding sequence along with the housekeeping genes *GAPDH* and *rRNA18S* (Applied Biosystem, Invitrogen, primers listed in Table 2). To compare the expression levels of the *BDNF* mRNA coding sequence between species, SYBR® master mix (Applied Biosystems, Invitrogen) was used together with the corresponding primers (Table 2). Relative *BDNF* gene expression levels were calculated using the 2^−ΔΔ*Ct*^ method and the housekeeping genes for normalization. The expression of proprotein convertases in rat Mks was analyzed using primers as described previously (17).

**TABLE 1:** **Primers used in conventional PCR experiments** m is mouse; r is rat; and h is human.

Name of oligonucleotide	Sequence (5′–3′)	Product size
mr *BdnfI* sense	GTGTGACCTGAGCAGTGGGCAAAGGA	803 bp
mr *BdnfII* sense	GGAAGTGGAAGAAACCGTCTAGAGCA	469 bp (IIa)
		682 bp (IIb)
		765 bp (IIc)
m *BdnfIII* sense	GCTTTCTATCATCCCTCCCCGAGAGT	425 bp
r *BdnfIII* sense	CCTTTCTATTTTCCCTCCCCGAGAGT	427 bp
mr *BdnfIV* sense	CTCTGCCTAGATCAAATGGAGCTTC	553 bp
mr *BdnfV* sense	CTCTGTGTAGTTTCATTGTGTGTTC	364 bp
m *BdnfVI* sense	GCTGGCTGTCGCACGGTTCCCATT	542 bp
r *BdnfVI* sense	GCTGGCTGTCGCACGGTCCCCATT	543 bp
mr *Bdnf* VII sense	CCTGAAAGGGTCTGCGGAACTCCA	420 bp
mr *Bdnf* VIII sense	GTGTGTGTCTCTGCGCCTCAGTGGA	362 bp
m *Bdnf* IXA sense	CCCAAAGCTGCTAAAGCGGGAGGAAG	
r *Bdnf* IXA sense	CCAGAGCTGCTAAAGTGGGAGGAAG	525 bp
mr *Bdnf* antisense	GAAGTGTACAAGTCCGCGTCCTTA	
h *BDNF* sense	GATGCCAGTTGCTTTGTCTTCTGTAG	471 bp
h *BDNFII* sense	GGGCGATAGGAGTCCATTCAGCACC	311 bp (IIa)
		526 bp (IIb)
		609 bp (IIc)
h *BDNFIII* sense	AGTTTCGGGCGCTGGCTTAGAG	346 bp
h *BDNFIV* sense	GCTGCAGAACAGAAGGAGTACA	411 bp
h *BDNFV* sense	TCGCGTTCGCAAGCTCCGTAGTG	272 bp (Va)
		282 bp (Vb)
		565 bp (V-VIII-VIIIh)
		682 bp (V-VIII)
h *BDNFVh* sense	GGCTGGAACACCCCTCGAA	339 bp
h *BDNFVI* sense	GGCTTTAATGAGACACCCACCGC	368 bp (VIa)
		386 bp (VIb)
		493 bp (VI-IXb)
h *BDNFVII* sense	GAACTGAAAGGGTCTGCGACACTCT	328 bp (VIIa)
		428 bp(VIIb)
h *BDNFIX* sense	TTTCTCGTGACAGCATGAGCAG	352 bp (IXabd)
		536 bp (IXabcd)
h *BDNF* antisense	GTCCTCATCCAACAGCTCTTCTATC	
hmr *HPRT* sense	GATGATGAACCAGGTTATGAC	469 bp
hmr *HPRT* antisense	GTCCTTTTCACCAGCAAGCTTG	

**TABLE 2:** **Primers with references used in real time quantitative PCR experiments** m is mouse; r is rat; and h is human.

Name of oligonucleotide	Reference or sequence (5′–3′)	Product size
m *Bdnf* (coding sequence)	Mm04230607_s1	92 bp
m *BdnfI*	Mm01334047_m1	105 bp
m *BdnfIV*	Mm00432069_m1	145 bp
m *BdnfVI*	Mm01334042_m1	108 bp
m *BdnfIXa*	Mm04230616_s1	77 bp
m *Gapdh*	Mm99999915_g1	109 bp
m *rRNA18S*	Mm03928990_g1	61 bp
r *Bdnf* (coding sequence)	Rn02531967_s1	142 bp
r *BdnfI*	Rn01484924_m1	106 bp
r *BdnfIV*	Rn01484927_m1	120 bp
r *BdnfVI*	Rn01484928_m1	109 bp
r *BdnfIXA*	Rn04230568_s1	95 bp
r *Gapdh*	Rn01775763_g1	174 bp
r *rRNA18S*	Rn03928990_g1	61 bp
h *BDNF* (coding sequence)	Hs02718934_s1	74 bp
h *BDNFI*	Hs00538277_m1	104 bp
h *BDNFIV*	Hs00380947_m1	116 bp
h *BDNFVIa*	Hs04188535_m1	119 bp
h *BDNFVIb*	Hs00156058_m1	143 bp
h *BDNFIXabcd*	Hs00542425_s1	81 bp
h *GAPDH*	Hs02758991_g1	93 bp
h *rRNA18S*	Hs03003631_g1	69 bp
mrh *BDNF* sense (coding sequence)	GAGCTGAGCGTGTGTGACAG	256 bp
mrh *BDNF* antisense (coding sequence)	CGCCAGCCAATTCTCTTTTTGC	
mrh *rRNA18S* sense	GTCTGTGATGCCCTTAGATG	176 bp
mrh *rRNA18S* antisense	AGCTTATGACCCGCACTTAC	

##### Statistical Analysis

All values are expressed as mean ± S.E. All statistical tests were paired *t* test, one-tailed.

## Results

**FIGURE 1.**
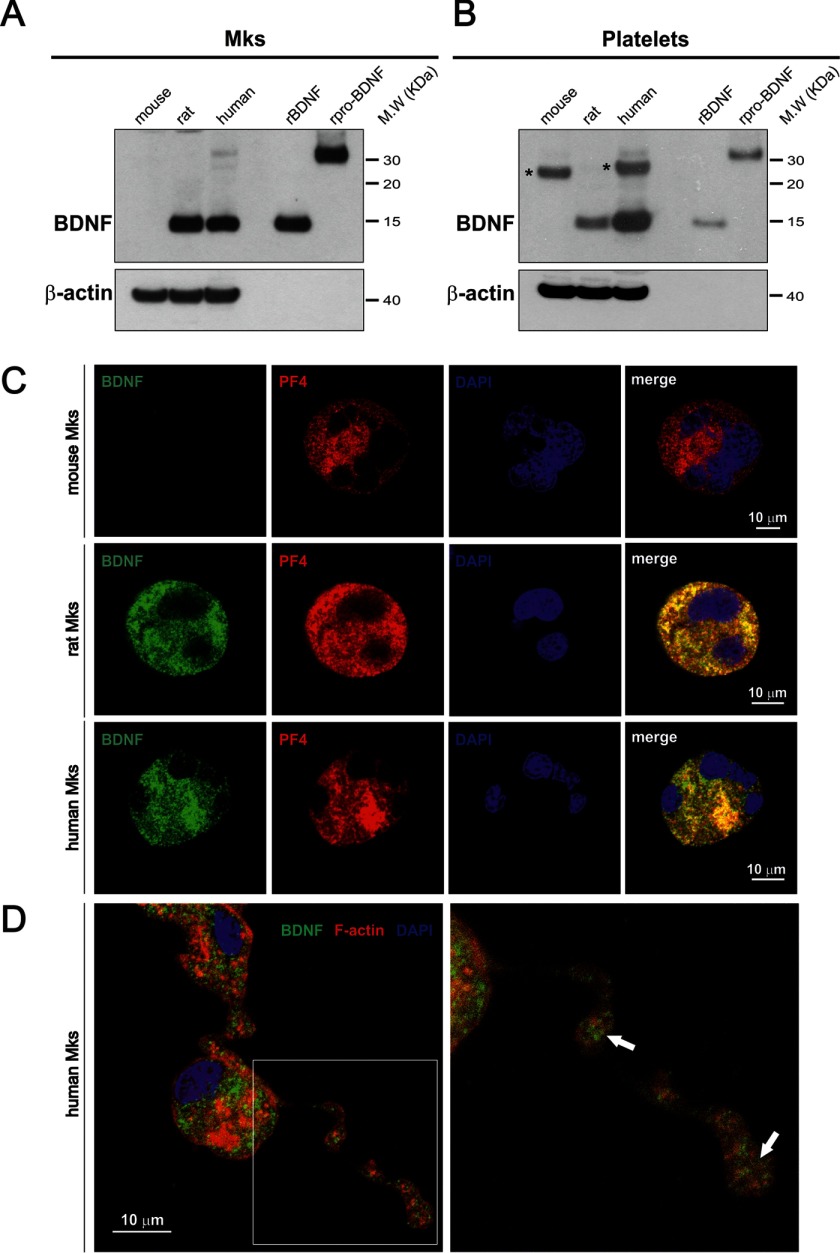
**Differential BDNF protein levels in mouse, rat, and human megakaryocytes and platelets.** Western blot lysates of cultured Mks (*A*) and blood platelets (*B*) are shown. Eighty micrograms of protein per lane were loaded, and the blotting membrane was incubated with the mouse monoclonal antibody 3C11 developed by Icosagen (Tartu, Estonia). Recombinant BDNF and pro-BDNF were used as molecular mass markers and antibodies to β-actin as loading controls. *Asterisks* (*top right panels*) point to a band unrelated to BDNF likely corresponding to immunoglobulin light chains in the mouse sample. Note the absence of BDNF in mouse Mks and platelets. *C,* antibodies to BDNF 9 (*green*) (15) and PF4 (*red*) reveal expression of both antigens in mature rat and human Mks. Note that unlike PF4, BDNF is not detectable in mouse Mks. The co-localization of BDNF with PF4 in rat and human Mks was quantified using the pixel intensity specifically generated by each channel. In humans, 83% and in rats 86% BDNF-positive granules were also PF4-positive. *Blue*, DAPI staining. *D,* immunofluorescence staining of F-actin (*red*) and BDNF (*green*) in proplatelet-forming cultured human Mks. *Arrows* indicate BDNF accumulation in proplatelet buds.

**FIGURE 2.**
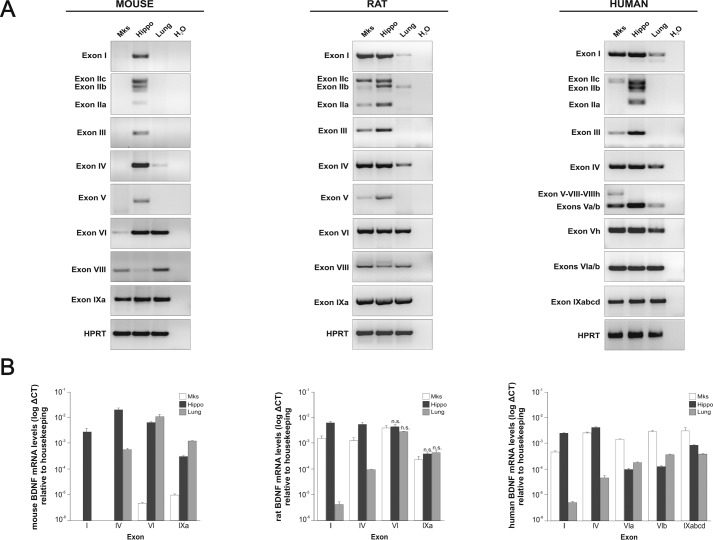
**Transcriptional analysis of *BDNF* in mouse, rat, and human megakaryocytes.** Conventional (*A*) and real time quantitative (*B*) PCR using exon-specific primers with RNA extracted from mature cultured Mks, adult hippocampus (*Hippo*), and lung are shown. Note that in the mouse, the neuron-specific transcripts, including exon I and IV, are not detected and that by contrast the transcript pattern resembles the non-neuronal pattern observed in lung tissue. The converse is the case with RNA extracted from rat and human Mks with transcript patterns, including exon I and IV, that are characteristic of a neuronal pattern as illustrated with the hippocampus. Unless indicated as non-significant (*n.s.*), all values are mean values ± S.E. in triplicates and based on three independent experiments, at *p* < 0.001 (paired *t* test).

To determine whether BDNF can be detected in Mks, we established primary cultures of mouse, rat, and human Mks and analyzed their content by Western blotting using a BDNF monoclonal antibody specifically recognizing BDNF and not NGF or NT4, under conditions where both are recognized by their corresponding antibodies (data not shown). A strong signal corresponding to the expected size of the monomer of mature BDNF was detected in both rat and human but not in mouse Mks (Fig. 1*A*). Note that pro-BDNF is barely detectable under these conditions, with only a faint band detected in human Mks. As a control, we also analyzed the BDNF content of platelets purified from the corresponding species and confirmed the presence of considerable amounts of BDNF in human platelets, with substantial levels also detected in rat but not in mouse platelets (Fig. 1*B*). This result with platelets is in agreement with previous conclusions using immunoassay determinations with the serum prepared from these three species (18). We then explored whether BDNF would be localized in α-granules, the storage compartment of a number of growth factors in the Mk lineage. A BDNF-specific signal was found to extensively co-localize with PF4, also designated CXCl4, one of the most abundant α-granule proteins (Fig. 1*C*). A distinct BDNF signal could also be seen in the tips of the proplatelet-forming human Mks (Fig. 1*D*, *arrows*), consistent with the notion that BDNF is transferred from Mks to platelets. As a control for the specificity of the BDNF signal in these immunostaining experiments, we used mouse Mks. When incubated with the corresponding antibodies under the same experimental conditions as rat and human Mks, PF4, but not BDNF, was detected (Fig. 1*C*). The lack of any detectable BDNF in mouse platelets is not a feature specific to CD1 mouse strain that we used in most of our experiments, as the C57BL/6 strain led to identically negative results, both in Mks and platelets. We note that in a recent highly sensitive and quantitative proteomic analysis of C57BL/6 platelet extracts, no BDNF could be detected (19). Quantification of the BDNF levels in rat Mks by ELISA indicated that their lysates contain 1.40 ± 0.13 ng/mg protein (mean ± S.E., *n* = 6). We also attempted to determine whether proplatelet-forming Mks release BDNF into the medium by incubating the cells with HRP-conjugated BDNF monoclonal antibodies (see “Experimental Procedures”). After 2 days of incubation with the BDNF capture antibody, we failed to detect any release of BDNF into the Mk-conditioned medium, suggesting that the bulk of BDNF is transferred into platelets and not released into the medium. This result is consistent with the previous work indicating that both in rats and humans the levels of BDNF are far higher in serum than in plasma, suggesting that the bulk of BDNF in serum results from platelet degranulation (18). To determine whether the BDNF biosynthetic machinery is expressed in Mks, we isolated RNA from mature platelet-forming mouse, rat, and human Mks. Significant levels of *BDNF* mRNAs were detected in both rat and in human cells (Fig. 2*A*). In four separate experiments, the total mRNA levels in both species were found to be about 200-fold higher in rat and human, compared with mouse Mks. All known *BDNF* transcripts present in RNA extracted from Mks of the three species were analyzed and compared with RNA extracted from the hippocampus and the lung as neuronal and non-neuronal reference tissues of the corresponding species (Fig. 2*A*). The results of these experiments revealed a neuronal pattern of mRNA expression in both rat and human Mks, with a prominent inclusion of exon I and IV transcripts. Notably, exon I-containing transcripts have recently been shown to markedly increase *Bdnf* mRNA translatability (20). By contrast, these typical neuronal transcripts were undetectable in the mouse (Fig. 2*A*). The levels of all main transcripts were also assessed in the three species by real time PCR (Fig. 2*B*).

**FIGURE 3.**
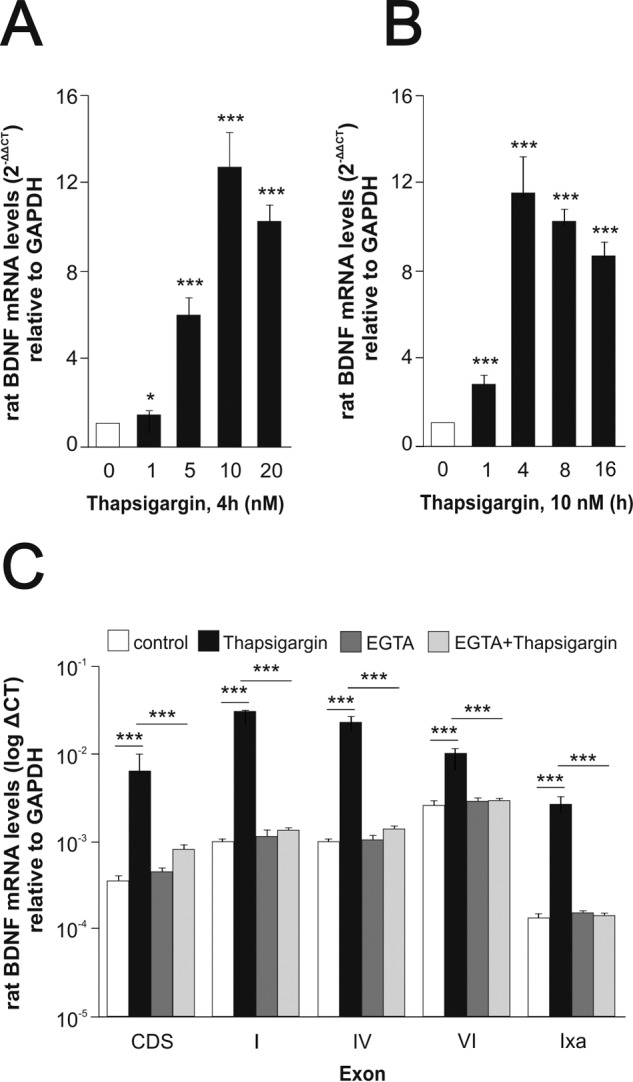
**Up-regulation of** Bdnf **mRNA by thapsigargin.** Effect of extracellular calcium. Dose response (*A*) and time course (*B*) of *Bdnf* mRNA expression by rat Mks after thapsigargin treatment. Purified mature rat Mks were cultured in the presence or absence of thapsigargin or vehicle (DMSO) used at the indicated concentrations (*A*) and for different lengths of times (*B*). Total mRNA was extracted and reverse-transcribed, and the resulting cDNA was amplified by real time quantitative PCR using specific primers for the coding sequence of *Bdnf. C,* extracellular calcium dependence of thapsigargin-induced *Bdnf* mRNA increase. Rat Mks were preincubated with 2.5 mm EGTA for 1.5 h at 37 °C followed by 10 nm thapsigargin for 4 h. mRNA expression was analyzed by real time quantitative PCR using specific primers for the coding sequence of *Bdnf* (*CDS*) or exon-specific primers. All values are mean values ± S.E. in triplicates and based on three independent experiments. Unless indicated, all the statistical values are compared with the control. *, *p* < 0.05; ***, *p* < 0.001 (paired *t* test).

**FIGURE 4.**
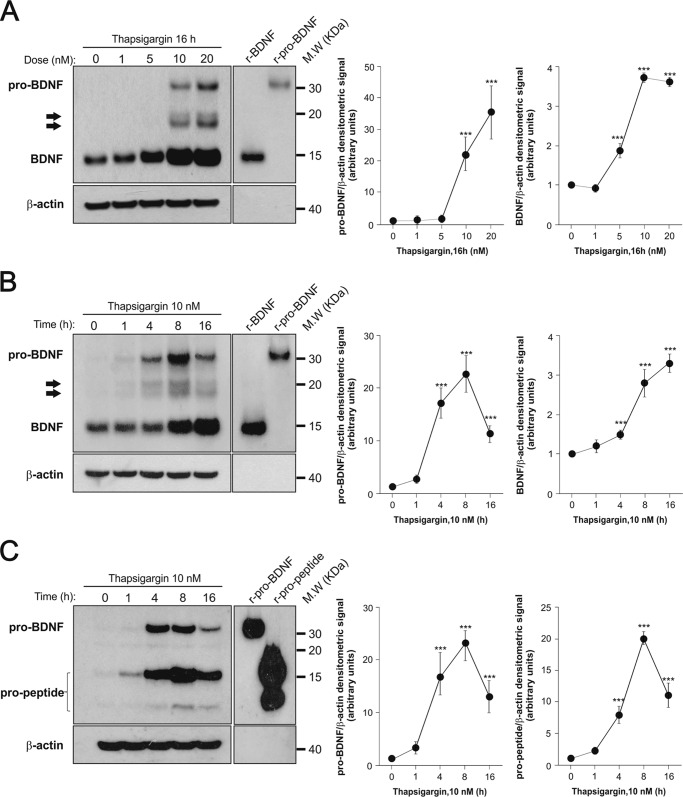
**Effect of thapsigargin on pro-BDNF, mature BDNF, and pro-peptide in rat Mks.** Dose response (*A*) and time course (*B*) of pro-BDNF and mature BDNF proteins by rat Mks after thapsigargin treatment are shown. Mature Mks were cultured for 16 h at the indicated doses of thapsigargin (*A*) or 10 nm thapsigargin for the indicated times (*B*). Forty micrograms of protein per lane were loaded, and the blotting membrane was incubated with the mouse monoclonal antibody 3C11 developed by Icosagen. *Arrows* indicate intermediate proteolytic products of pro-BDNF (*C*). Time course of pro-BDNF and pro-peptide proteins generated by rat Mks incubated with 10 nm thapsigargin for the indicated time periods. Eighty micrograms protein per lane were loaded, and the blotting membrane was incubated with the mouse monoclonal antibody H1001G developed by GeneCopeia, Inc. The blots shown are representative of three independent experiments with similar results. *Graphs* show mean ± S.E. of the densitometric values quantified from the blots of the three separate experiments. ***, *p* < 0.001 (paired *t* test compared the corresponding controls). Recombinant BDNF (150–300 pg), cleavage-resistant recombinant pro-BDNF (0.5–1 ng), and recombinant pro-peptide (1–10 ng) were used as molecular mass markers and antibodies to β-actin as loading controls.

**FIGURE 5.**
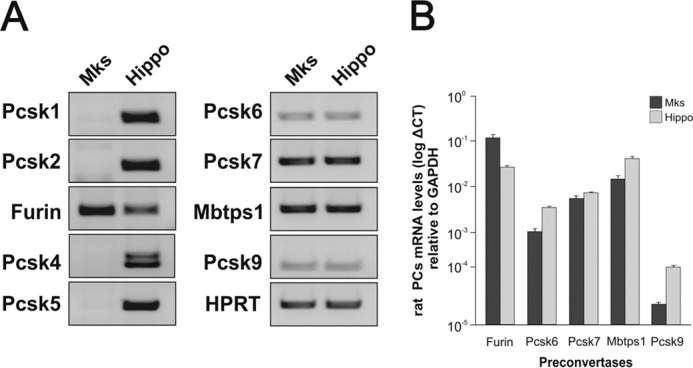
**Differential expression of proprotein convertases in primary MKs.** Conventional (*A*) and real time quantitative (*B*) PCRs using specific primers and RNA extracted from mature cultured rat Mks and adult hippocampus are shown. Note that although transcripts, including *Pcsk1, PcsK2, Pcsk4,* and *Pcsk5*, are expressed in hippocampal tissue, they are not detected in Mks (*A*). Comparative expression levels between the two tissues for the expressed proprotein convertases are shown in *B*. All values are mean values ± S.E. in triplicates and are based on three independent experiments.

In neurons, increased levels of cytoplasmic calcium have long been known to activate *Bdnf* transcription by activating promoters I and IV in particular (21). As Mks are devoid of voltage-activated calcium channels, the addition of thapsigargin to non-electrically excitable cells such as Mks offered an opportunity to test whether the corresponding *Bdnf* promoters are also responsive to calcium levels in Mks. Thapsigargin is a selective inhibitor of the sarco/endoplasmic reticulum Ca^2+^-ATPase, secondarily leading to the opening of stored-operated calcium channels at the cell surface and increased levels of cytoplasmic calcium (22). We found that at nanomolar concentrations (Fig. 3*A*), thapsigargin massively activates transcription in a time-dependent manner (Fig. 3*B*). This increase was primarily accounted for by contributions from exons I, IV, and IXa and to a lesser extent exon VI (Fig. 3*C*). We then tested whether complexing extracellular calcium would decrease the thapsigargin-induced transcriptional activation of *Bdnf* and found that 2.5 mm EGTA completely blocked the inductive effects of thapsigargin as assessed both by primers corresponding to the protein coding sequence or by exon-specific primers (Fig. 3*C*). To further test the notion that intracellular calcium levels regulate *Bdnf* transcription, we tested the effects of the calcium ionophore ionomycin. When added to mature Mk cultures for 4 h, it increased the levels of *Bdnf* mRNA by 10.50 ± 1.85-fold (*n* = 3, mean ± S.E.). As still very little is known about the biosynthesis of endogenous BDNF in any cell type, we were then curious to see how the Mk translation and processing machinery would cope with the massive increase in *Bdnf* mRNA levels caused by thapsigargin. We found that at 10 nm, thapsigargin led not only to a marked increase of processed (or mature) BDNF but also to readily detectable levels of pro-BDNF, suggesting that the thapsigargin-induced increased transcription may temporarily saturate the pro-BDNF processing capacity of Mks (Fig. 4, *A–C*). The 3C11 BDNF monoclonal antibody recognizes not only mature but also (as expected) unprocessed and partially processed forms of BDNF as indicated in Fig. 4, *A* and *B*. In previous experiments using heterologous expression systems, we noted that the replacement of an arginine residue in position −1 by lysine at the furin cleavage site of pro-BDNF led to the accumulation of an *N*-glycosylated intermediate product corresponding to the size indicated by the *arrows* in Fig. 4, *A* and *B*. Amino-terminal sequencing of this product indicated that the use of an alternative cleavage site generated a product with a 15-residue addition to the amino terminus of mature BDNF (23). This product was shown to be *N*-glycosylated (21). The use of the pro-BDNF monoclonal antibody H1001G independently confirmed the identity of pro-BDNF and also allowed the detection of the BDNF cleaved pro-peptide (Fig. 4*C*). We note that in the absence of thapsigargin stimulation, the steady state levels of pro-BDNF are even lower than in neurons where we estimated them to represent about 1 molecule for 10 molecules of mature BDNF (8, 15). We also tested the expression of the most likely protease candidate thought to be involved in the generation of mature BDNF, namely furin and the proprotein convertase 7, the latter having been recently shown to be necessary for the processing of BDNF in neurons (24). The results of conventional (Fig. 5*A*) and real time quantitative PCR (Fig. 5*B*) experiments indicated that both enzymes are present in rat Mks, at levels comparable with those found in the rat hippocampus (Fig. 5). In line with this, the processing of pro-BDNF was largely prevented by the addition of the convertase inhibitor decanoyl-Arg-Val-Lys-Arg-chloromethyl ketone (data not shown).

## Discussion

The results obtained with primary cultures of Mks suggest that these cells represent the main source of BDNF in platelets as well as in serum. First, Mks contain readily detectable levels of BDNF protein. Second, BDNF is stored in α-granules in Mks, long known to also represent the storage compartment of various growth factors and cytokines in platelets (25). Third, BDNF can be visualized in proplatelets (Fig. 1*D*) suggesting that platelets contain BDNF by the time they begin to separate from Mks. Fourth, the *BDNF* gene is expressed at relatively high levels in rat and human Mks, although by contrast, the levels are much lower in mice. Notably, the mouse transcripts do not include exon I, which allows efficient translation of BDNF mRNA (20). This negative result with mouse Mks is significant as it correlates with the lack of detectable levels of BDNF in mouse serum, unlike the case with rat and human sera (18). Taken together, it would seem that circulating platelets, once filled up with BDNF packaged in α-granules inherited from Mks, represent the only significant source of BDNF in serum. Other sources such as endothelial or immune cells that do express the *BDNF* gene at low levels (26, 27) do not seem to make significant contributions to circulating levels of BDNF as the levels are undetectable in mouse serum (18). Also, the very low levels of BDNF found in human and rat plasma may in fact be accounted for by microparticles or exosomes released from platelets (28). It is intriguing to note that human platelets and sera contain BDNF levels that are about 10 times higher than in the rat. The reasons for this difference are unclear at this point, as is the function of BDNF in platelets. The lack of BDNF in mouse platelets suggests that whatever the biological role of platelet-derived BDNF may be, it could be redundant in the mouse with the function of other platelet-derived growth factors. A signaling system based on the circulation of small and ubiquitous cellular fragments, including exosomes loaded up with a powerful neurotrophic factor, has the potential to be functionally relevant in the context of human brain function. As blood flow is tightly regulated by neuronal activity (29), it is conceivable that in humans exosomes loaded with BDNF (30) may be delivered to the brain in activity-dependent fashion, possibly explaining the beneficial effects of physical exercise. Although this is a matter of speculation at this point, the possibility of a functional role for BDNF in platelets can now be tested by engineering the mouse genome so as to replicate the situation in humans. Alternatively, it is also conceivable that the functional significance of BDNF in human platelets may remain as mysterious as that of NGF and EGF in the adult male mouse submandibular gland (31).

It may seem surprising that the cellular source of BDNF in rat and human serum has not been previously uncovered, especially in view of the very extensive use of BDNF as a biomarker in human blood (32). An analysis of the corresponding literature reveals that negative results were obtained early on in experiments specifically addressing the question of BDNF expression in human Mks (16). These experiments were performed with the megakaryocyte lines DAMI and Meg-01 and led to the conclusion that the *BDNF* gene is not expressed in the cells (16). Although we confirmed these results, it appears plausible that these tumor lines fail to faithfully replicate late aspects of Mk maturation, as is not rarely the case with readily expandable tumor cells. Following this negative result, the presence of BDNF in platelets has been speculated to results from a hypothetical uptake from sources such as the brain. However, this notion has not been substantiated by plausible mechanisms, unlike in the case of serotonin, a neurotransmitter long known to accumulate in the dense granules of platelets following its uptake by specific transporters located in the membrane of platelets.

The identification of Mks as the source of BDNF in platelets invites a revision of the widely held view that in humans the serum levels of BDNF reflect its levels in the brain. In addition to our findings, it has long been established that radiolabeled BDNF does not reach the brain when injected into the peripheral circulation (33). It appears then that the variations in the levels of BDNF reported in various conditions, including the increase after physical exercise (14) or decrease during the course of depressive episodes (13), are in need of alternative plausible explanations, and it is conceivable that these variations may reflect different degrees of platelet activation (34). In addition, there is emerging evidence that the hematopoietic niche where Mks develop (35) is innervated by the peripheral nervous system and that hematopoietic cells may respond to nerve-derived signals (36). However, whether or not these stimuli change the expression levels of BDNF in Mks remains unclear at this point.

With regard to the biosynthesis of endogenous BDNF, our results suggest that Mks could represent an alternative cellular model to neurons, which have been so difficult to study in the face of the very low levels of expression of BDNF levels in these cells. By comparison with BDNF levels in the brain (11), the levels of BDNF in human platelets are significantly higher, 100–1,000-fold on a per mg of protein basis when brain extracts and purified platelets are compared (12). In particular, Mks offer a new opportunity to examine the biosynthesis of endogenous BDNF and the possible role of pro-BDNF. Transcription activation by thapsigargin leads to clearly detectable levels of pro-BDNF without the need for prior enrichment by immunoprecipitation. In view of the current interest related to the Val → Met substitution in pro-BDNF (3), human Mks of the corresponding genotype may represent an interesting cellular model to understand the biochemical consequences of this amino acid replacement.

In conclusion, our results contribute to clarify the cellular origin of BDNF in human blood; and they describe a tractable cellular system to study the biosynthesis of endogenous BDNF.

## Author Contributions

P. C. F. designed, performed, and analyzed the experiments illustrated in Figs. 1–5 and K. S. was also involved. T. M., M. C., and C. G. were involved in all aspects of the work related to human, rat, and mouse Mks and in the interpretation of the results. Y. A. B. helped with the initiation of the project, the design of the experiments, and with the interpretation of the results. All have read the manuscript and discussed its content. The final version was approved by all.

## Footnotes

2 The abbreviations used are:

BDNF: brain-derived neurotrophic factor
TPO: thrombopoietin
TRITC: tetramethylrhodamine isothiocyanate
Mk: megakaryocyte.
